# Supplementary material for: Decreased accuracy of erythrocyte sedimentation rate in diagnosing osteomyelitis in diabetic foot infection patients with severe renal impairment: A retrospective cross-sectional study
Source: PLoS One. 2022 Mar 23;17(3):e0265769. doi: 10.1371/journal.pone.0265769 (PMC8942204; doi:10.1371/journal.pone.0265769)
Supplement: Attachment — Submitted filename: Response to Reviewers.docx [file pone.0265769.s003.docx]

Dear Ph.D Kanhaiya Singh and reviewers:

Thank you for your letter and for the reviewers’ comments concerning our manuscript entitled “Decreased accuracy of erythrocyte sedimentation rate in diagnosing osteomyelitis in diabetic foot infection patients with severe renal impairment: a retrospective cross-sectional study” (ID: PONE-D-21-37187). Those comments are all valuable and very helpful for revising and improving our paper, as well as the important guiding significance to our research. We have studied comments carefully and have made correction which we hope meet with approval. Revised portion are marked in red in the paper. The main corrections in the paper and the responds to the reviewer’s comments are as flowing:

Responds to the reviewers’ comments:

Reviewer #1:

**1）Response to comment**: Abstract: The starting paragraph is not scientifically developed. If there is already plethora of studies, why authors want to study this? There is no gap of knowledge is shown.

**Response:** Thank you for your reminding. We are sorry for neglecting to introduce knowledge gap in Abstract. We have added background section according to the reviewer’s suggestion.

**2) Response to comment:** 1. Inclusion criteria: The authors have studied patients with severe renal impairment: No mention has been made of patients receiving Hemodialysis/ peritoneal dialysis for the diabetic nephropathy. As noted in a previous study by Alisomali MI et al (ref no 29): Post-dialysis the ESR was raised in most of the stable patients on regular HD and was significantly higher than the pre-dialysis ESR (by, on average, 5.8 mm/h).

**Response:** We feel great thanks for your professional review work on our paper. As you are concerned, the weakness needs to be acknowledged. Limited by retrospective approach, we could only had obtained data from morning fasting blood test, and no control data 1 hour after hemodialysis. According to the study by Alisomali MI et al, their study only included stable hemodialysis patients, and patients with active infection were excluded. We believe that the main reason for the increase of ESR at 1 hour after dialysis might be the inflammatory response induced by hemodialysis itself. When infection become the main reason for elevated ESR, the influence of blood collection time on ESR still needs further investigation. We have added the number of patients receiving hemodialysis among the included cases, and have written above concern in the fourth limitation.

**3) Response to comment:** 2. Novelty concern and Study Design concerns: The same study (ref no 29) and several other studies (cited below) have mentioned that 'high ESR may be limited diagnostic utility in patients with Chronic kidney disease'. Making the entire diagnostic value in patients specifically having Diabetic foot infection and osteomyelitis redundant.

**Response:** We would like to thank you for your critical comment. The evidence gained here could help to understand the role of SRI in the diagnosis of DFO by ESR. In the studies mentioned by you, the included subjects were all stable dialysis patients, which means that subjects with recent infection were excluded. According to the mentioned literatures, the basal ESR level is increased in stable dialysis patients. However, these literatures lack control data in the infected state. When infection becomes main cause of elevated ESR, differences in ESR between dialysis patients and non-dialysis patients have not been reported before. Based on our data set, no statistically significant difference in ESR was found between SRI patients with and without dialysis, suggesting that dialysis-related ESR elevation may no longer predominate in patients with current infection (SRI patients with dialysis, 68±28 mm/h; SRI patients without dialysis, 67±26 mm/h, *P* = 0.919, Kruksal–Wallis *H* test). Our study provided a deeper insight into the relationship between DFI and SRI, and contributed to the understanding of the diagnosis of DFO by ESR.

**4) Response to comment:** As the authors mention later in study limitations, the diagnosis of Diabetic foot osteomyelitis has not been established by bone biopsy in majority which is a gold standard. (line 251)

**Response:** Thank you for your valuable comment. As you are concerned, the limitation needs to be noted. According to 2015 International Working Group on the Diabetic Foot guideline, bone biopsy is usually required only when the diagnosis is in doubt or determining the causative pathogen’s antibiotic susceptibility. It is also stated that bone biopsy would be theoretically useful to obtain a bone specimen in almost all cases, but it is often impractical as the procedure requires some time, experience, and expense in 2019 update guideline. For clinical practice, current reference standard could guide diagnosis and treatment well. Nevertheless, for diagnostic accuracy test, it is possible that DFO is misdiagnosed. Therefore, developing a reference standard that takes into account both diagnostic accuracy and clinical utility still requires further exploration. We have rewritten the third limitation to make easier for readers to understand.

**5) Response to comment:** Fig 2 'The difference in ESR between DFI patients with and without SRI': Individual data points could be shown in the Box-Whisker plot to compare two variables. Also the legend does not mention the type of graph or analysis used (Kruksal–Wallis H test)

**Response:** Thanks for your valuable comment. We have redrawn Fig 2 and rewritten legend based on reviewer’s suggestion.

**6) Response to comment:** Fig 3: more could have been done with the present data if authors could find the Positive and negative predictive value using ROC curves and prevalence of disease in population.

**Response:** Thanks for your constructive comment. We have calculated sensitivity, specificity, prevalence, positive predictive value, negative predictive value, positive likelihood ratio, and negative likelihood ratio with their 95% confidence intervals to make our paper more informative and comprehensive.

Special thanks to you for your good comments.

Reviewer #2:

**1) Response to comment:** The role of ESR in making a diagnosis of osteomyelitis in diabetic foot ulcers is complimentary to other investigations including imaging and bone scan. This is an interesting study stating that the adjunctive value of ESR in diagnosing osteomyelitis diminishes in patients with severe renal impairment. ESR may therefore be used as a screening test for making a presumptive diagnosis of osteomyelitis in patients with diabetic foot infections without renal impairment.

**Response:** Thanks for your careful reading of our manuscript. Based on the comments from other reviewers, we have modified some content to make our paper more in-depth and reasonable.

Special thanks to you for your good comment.

Reviewer #3:

**1) Response to comment:** 1. In line number 71, authors mentioned there is a high chance of diabetic patients to suffer from kidney diseases. But Fig. 1 shows out of 364 DFI patients only 44 are having SRI. Also, HbA1c% of patients with SRI is lower than patients who do not have SRI, as indicated in Table 1. Both these data are contradicting to the statement made in line number 7.

**Response:** Thanks for your careful checks. We have confirmed the accuracy of the mentioned data. Based on the data in S2 file, 111 patients in this study have underlying chronic kidney disease (eGFR < 60 mL/min/1.73 m^2^), a prevalence of 30.5% is similar to prevalence of chronic kidney disease in the Shanghai urban area reported in ref. 16 (29.6%–32.8%). As far as the patients with SRI have lower HbA_1c_, this may be related to the fact that these patients pay more attention to blood sugar control. When the disease is not severe, patients may ignore blood sugar management, resulting in a higher HbA_1c_ levels in patients without SRI.

**2) Response to comment:** 2. Conclusion part should be rewritten for clarity (refer to line 257). In line 264, no need to write the values in bracket as it is already mentioned.

**Response:** Thanks for your instructive comment. We have rewritten the conclusion part to make it well-structured and clear.

**3) Response to comment:** 3. Some graphs/diagrams corresponding to the statistical data table should be great to visualize the important parameters.

**Response:** Thanks for your valuable comment. Combined with suggestions from other reviewer, we have redrawn Fig. 2 and added Fig. 4 to provide more information.

**4) Response to comment:** 4. A list of abbreviations will be helpful for the readers, as there are too many abbreviations used.

**Response:** Thanks for your beneficial comment. We have added a list of abbreviations at the end of our paper.

**5) Response to comment:** 5. As the authors declare, "a prospective study with a priori calculated sample size is needed to verify the reliability of our conclusions" - it should be justified the relevance of study with the present data set with a future scope of research.

**Response:** Thanks for your helpful comment. We have added an explanation of relevance of the current findings and future research in the conclusion section.

Special thanks to you for your good comments.

Other changes:

1. We have added details about the excluded subjects in results part.

We tried our best to improve the manuscript and made some changes in the manuscript. These changes will not influence the content and framework of the paper. And here we did not list the changes but marked in red in revised paper.

We appreciate for Ph.D Kanhaiya Singh, and reviewers’ warm work earnestly, and hope that the correction will meet with approval.

Once again, thank you very much for your comments and suggestions.

Best regards,

Corresponding author:

Huafa Que

E-mail: huafaque@126.com
