# Supplementary material for: Decreased accuracy of erythrocyte sedimentation rate in diagnosing osteomyelitis in diabetic foot infection patients with severe renal impairment: A retrospective cross-sectional study
Source: PLoS One. 2022 Mar 23;17(3):e0265769. doi: 10.1371/journal.pone.0265769 (PMC8942204; doi:10.1371/journal.pone.0265769)
Supplement: Attachment — Submitted filename: Response to Reviewers.docx [file pone.0265769.s004.docx]

Dear Ph.D Kanhaiya Singh and reviewers:

Thank you for your letter and for the reviewers’ comments concerning our manuscript entitled “Decreased accuracy of erythrocyte sedimentation rate in diagnosing osteomyelitis in diabetic foot infection patients with severe renal impairment: a retrospective cross-sectional study” (ID: PONE-D-21-37187R1). Those comments are all valuable and helpful for improving our paper to make it easier to read and understand. We have studied comments carefully and have made correction which we hope meet with approval. Revised portion are marked in red in the paper. The corrections in the paper and the responds to the reviewer’s comments are as flowing:

Responds to the reviewers’ comments:

Reviewer #1:

**1）Response to comment**: Legends: lines 234-239: the authors may add some interpretations of the graph along with the methods used.

**Response:** Thank you for your valuable comment. We are sorry for neglecting to write figure legends. We have added figure legend section according to the reviewer’s suggestion.

**2) Response to comment:** Figure 4: the font needs to be unbolded so the image is easier to interpret.

**Response:** Thank you for your reminding. We have made the font in fig.4 unbolded, and also the font in fig.2 and fig.3 unbolded for unity.

Special thanks to you for your good comments.

Other changes:

1. We have re-uploaded Fig 1 to Fig 4 to comply with PLOS requirements.

We tried our best to improve the manuscript and made some changes in the manuscript. These changes will not influence the content and framework of the paper. And here we did not list the changes but marked in red in revised paper.

We appreciate for Ph.D Kanhaiya Singh, and reviewers’ warm work earnestly, and hope that the correction will meet with approval.

Once again, thank you very much for your comments and suggestions.

Best regards,

Corresponding author:

Huafa Que

E-mail: huafaque@126.com
